# First-year Medical Students’ Varying Vulnerability to Developing Depressive Symptoms and Its Predictors: a Latent Profile Analysis

**DOI:** 10.1007/s40596-023-01757-x

**Published:** 2023-03-01

**Authors:** Sabine Polujanski, Thomas Rotthoff, Ulrike Nett, Ann-Kathrin Schindler

**Affiliations:** University of Augsburg, Augsburg, Germany

**Keywords:** Medical students, Depression, Mental health, COVID-19, Protective factors

## Abstract

**Objective:**

Previous meta-analytic data have demonstrated the propensity for mental morbidity among medical students (Rotenstein et al. JAMA. 2016;316(21):2214–36). However, there is a lack of research on medical students’ varying depression vulnerabilities and predictive factors. The present study aims to gain a better understanding of the development of mental health morbidity and its predictive factors among first-semester medical students.

**Methods:**

In November 2020 and January 2021, 184 first-semester students from two medical schools were surveyed regarding depression (PHQ-9), self-efficacy, resilience, and cognitive self-regulation. Using latent profile analysis, we identified distinct depression development profiles. We applied a multinomial logistic regression analysis to determine how self-efficacy, resilience, and cognitive self-regulation and their changes predicted profile membership.

**Results:**

Five profiles of depression development were identified: profile 1, no depression (53.8%); profile 2, mild depression (26.1%); profile 3, depression increase I (9.2%); profile 4, depression increase II (9.8%); and profile 5, persistent depression (1.1%). Students with initially high self-efficacy, resilience, and cognitive self-regulation levels were more likely to belong to the no depression profile. A decrease in self-efficacy and cognitive self-regulation was associated with both depression increase profiles (profiles 3 and 4), and a decrease in resilience was found to be a predictor of profile 4.

**Conclusion:**

Students who enter medical school have varying states of mental health, and they differ in their vulnerability to developing depressive symptoms. The promotion of resilience, self-efficacy, and cognitive self-regulation strategies may be key in preventing students’ depression in the first semester of medical school.

Depression among physicians has been internationally reported [1], affecting patient safety due to a higher probability of medical errors [2]. A higher propensity for medical students to develop depressive symptoms has already been described [3]. An international meta-analysis demonstrated that nearly one-third of medical students (27%) reported depressive symptoms or depression [3]. Since medical students need to be mentally healthy to provide healthcare in the future, the need for depression prevention in medical schools is undeniable.

The first year of medical school appears to contribute to the development of mental morbidity [4]. It is assumed that the confrontation with unfamiliar university learning practices, habituation to examinations, developing new relationships, and changes in life circumstances that often accompany the start of studies (e.g., moving to a new city) [5] are external stressors affecting all freshmen and may lead to mental morbidity. In addition, the COVID-19 pandemic may have further impeded this life transition, as suggested by a US university study: 71% of (non-medical) students reported increased stress levels after 1 month of a stay-at-home order (April 2020) [6]. Additionally, a representative German (*N* = 3382) study conducted in July 2020 revealed clinically relevant depressive symptoms in 37% of the participating university students [7]. Likewise, a study conducted in May and June 2021 found in 42% of the participants an indication of major depression [8]. In both studies, medical students were less affected [7, 8]. The reason could be that, because of their involvement in the health system, medical students had more opportunities for social interaction compared with other fields of study [8] and that some practical parts of the curriculum could not be relocated and took place regularly.

Based on the transactional model of stress and coping [9], we assumed that not all medical students are equally vulnerable to developing depressive symptoms. According to this model [9], mental health can be understood as an interaction between the evaluation of *external* conditions (e.g., study conditions) and *internal* personal factors (e.g., available coping strategies). To date, few longitudinal studies have differentially examined changes in mental health. To the best of our knowledge, only one previous study has used person-centered methodology to investigate medical students’ varying mental health changes measured by depression during the course of their studies, as well as associated factors [10]. Person-centered approaches such as latent profile analysis are used to identify latent subgroups within a sample based on response behavior [11] and are considered beneficial for the design of preventive measures [12]. Unlike variable-centered approaches (i.e., correlation and regression analyses) that focus on the relationships between variables, person-centered methodologies consider individual differences. While the topic of physicians’ mental health has become part of international standards, there is still a poor understanding of how differential preventive measures might look like. Silva et al. [10] examined three patterns of depression development in medical students using the k-means clustering method: (1) students who did not develop any depression; (2) students with initial elevated depression values remaining persistent; and (3) students with initial elevated depression values experiencing recovery during medical school. The students in the persistent depression group tended to choose medical studies primarily because of expected income and prestige, whereas those in the recovery group chose medical studies because of their interest in the medical profession itself. These students experienced fewer learning problems and higher satisfaction with social activities.

Following this study, we were interested in exploring protective factors that could be promoted by medical schools. Previous studies have suggested that depression in general is associated with multiple demographic, social, and behavioral factors, such as age, economic situation, and family-related problems [13, 14]. Among university students, multiple academic factors can contribute to the development of depressive symptoms. A previous literature review found that such factors included false expectations regarding the curriculum and workload, high workload pressure (e.g., examinations), fear of poor grades, and unfavorable relationships with teachers [14]. There is mixed evidence on whether medical students are more affected by depression than their non-medical peers [15].

Individual protective characteristics, which are promotable by medical schools, can ameliorate the negative effects of the described stressors. Higher *self-efficacy* is associated with lower mental distress [16], and self-efficacy-based interventions can have a positive effect on mental health [17]. Moreover, higher levels of *resilience* are associated with lower levels of stress [18]. Resilience interventions have been shown to be effective in reducing depressive symptoms [19], and some findings suggest that resilience among medical students (i.e., measured by the mean value of the entire sample) gradually decreases during the first year of study [20]. In addition, there is also evidence that *cognitive self-regulation* can help individuals cope with high demands and have a positive impact on well-being [21]. *Cognitive self-regulation* includes strategies such as goal setting, planning, monitoring, and self-reflection, which are useful in coping with a large amount of learning material [22] — a major academic stressor in medical school [14]. However, cognitive self-regulation strategies may need to be adapted to suit new requirements in the first year of medical school. In medical students, these constructs have not yet been sufficiently studied in terms of different entry conditions and changes over time. This may be due to the misassumption that medical students belong to high-performing groups of students who do not need support. 

This study was planned under regular conditions before the COVID-19 pandemic, and its first objective was to screen for first-semester medical students’ depression and prevalence rates. However, because of the outbreak of the pandemic, classes during the investigated semester took place predominantly online. Thus, we also decided to compare our results with pre-pandemic studies to provide a better understanding of the effects of COVID-19-related virtual learning.

The second objective of this study was to identify distinct depression development profiles by performing a latent profile analysis (LPA) to understand the differing changes in mental health during the first semester of medical school. Thus, we provide a methodological added value for the differential examination of changes in mental health which may also be beneficial for other populations at a high risk of mental morbidity.

The third objective of this study was to investigate whether self-efficacy, resilience, cognitive self-regulation, and changes in these constructs predict latent profile membership. The findings could provide important complementary insights into students’ differing needs, which should be considered when designing preventive measures.

## Methods

### Study Design

This longitudinal study was conducted at two German medical schools (centers 1 and 2) at two measurement points. The participants responded to online questionnaires in the first week of medical school (T1: November 2020) and at the end of the first semester, 4 weeks before the examination period (T2: January 2021). The curricula of both medical schools are characterized by an integration of biomedical basics and clinical content. During the first semester, students are involved in lectures, seminars, bedside teaching, tutorial groups, and skills training. Unexpectedly, because of the COVID-19 pandemic, the investigated semester started 2 weeks later than regular and took place mainly online. Bedside teaching and skills training took place regularly only if ensured by the pandemic situation. Participation in the study was voluntary. Pseudonymized identification codes were used for the longitudinal allocation of the data.

### Measures

The personal data collected included gender, age, university entrance qualification grade, waiting period before entering medical school, and previous university experience.

#### Depression Severity

We applied the Patient Health Questionnaire-9 (PHQ-9) [23] (German translation [24]) to assess depression severity, which included nine items (*α*_T1_ = .81; *α*_T2_ = .84). Each item (e.g., “little interest or pleasure in doing things”) of the screening instrument was answered on a 4-point Likert scale ranging from 0 (*not at all*) to 3 (*nearly every day*). Each item corresponds to one of the nine DSM-IV diagnostic criteria for major depression. For the PHQ-9 items, a summative score less than 5 indicates no depression, 5–9 indicates mild depression, 10–14 indicates moderate depression, 15–19 indicates moderately severe depression, and 20–27 indicates severe depression. PHQ-9 scores of 10 points or above are associated with a specificity of 88% and a sensitivity of 88% for major depressive disorders. Thus, a PHQ-9 score of 10 is considered a clinically relevant cut-off value [23].

#### Self-efficacy

Perceived self-efficacy (*α*_T1_ = .82; *α*_T2_ = .87) was measured using the General Self-Efficacy Scale [25]. This 10-item scale (e.g., “I can always manage to solve difficult problems if I try hard enough”) is scored on a 4-point Likert scale from 0 (*not at all true*) to 3 (*exactly true*).

#### Resilience

Resilience (*α*_T1_ = .77; *α*_T2_ = .84) was assessed using the short version (CD-RISC-10 [26]) of the Connor-Davidson Resilience Scale (CD-RISC) [27]. This 10-item scale (e.g., “I can deal with whatever comes”) is scored on a 5-point Likert scale from 0 (*not true at all*) to 4 (*true nearly all the time*).

#### Cognitive Self-regulation

Cognitive self-regulation (*α*_T1_ = .72; *α*_T2_ = .77) was assessed using 11 items (e.g., “Before I start an extensive task, I determine how I will proceed.”) [21] scored on a 5-point Likert scale from 0 (*strongly disagree*) to 4 (*strongly agree*).

### Data Analysis

We included only data from complete responses by the students who participated at both measurement points. We applied IBM SPSS Statistics 26 to conduct preliminary analyses (i.e., descriptive statistics and correlation analyses), dropout analyses (i.e., *t*-tests and chi-square tests), and center analyses (i.e., *t*-tests) and to examine overall changes in depression (paired sample *t*-test) and prevalence rates (i.e., frequencies).

To determine the number of depression change profiles and the prevalence of each, a latent profile analysis (LPA) was conducted using M*plus* 8.2 [28]. LPA is a categorical latent variable modeling method used to identify latent subgroups in a sample based on response behavior [11]. To avoid local maxima, 400 random starts were used. The sample-adjusted Bayesian information criteria (SABIC), bootstrapped likelihood ratio tests (BLRT), Lo-Mendell-Rubin test (LRT), Akaike information criterion (AIC), Bayesian information criterion (BIC), and entropy levels were applied to examine the appropriate number of profiles. Lower SABIC, AIC, and BIC levels and higher entropy levels indicate better profile solutions [11, 29]. The LRT and BLRT indices indicate whether the N-class solution fits the observed data significantly better than the N-1-class solution [11, 29]. If multiple potential profile solutions are suggested by the fit indices, a decision regarding the best solution is recommended based on theoretical interpretability [30].

After the number of profiles was determined, we examined the associations between profile membership and the hypothesized predictors (i.e., self-efficacy, resilience, cognitive self-regulation, and their changes) by conducting a series of multinomial logistic regression analyses using the automated three-step procedure (R3STEP-command) [31]. Standardized predictors were entered separately into the regression analyses. For small profile sizes, further effects were interpreted with caution.

## Results

### Participants

A total of 296 participants responded to the questionnaire at T1. Of those, 184 students (center 1: *n* = 87; center 2: *n* = 97) completed the questionnaire at T2 (dropout rate = 38%, *n* = 112). Only students who participated in the survey twice were included in the analyses. At T1, 71% of the participants were female, and 29% were male (0% nonbinary). The mean age was 21.95 years (*SD* = 3.20). About half of the students (49.5%, *n* = 91) reported a waiting period before starting medical school of 3.68 years (*SD* = 2.83; *n* = 87). The mean university entrance qualification grade was 1.56 (*SD* = 0.57, *n* = 183). In the German grading system, A-level grades range from 1.0 to 4.0 (1.0 = very good, 2.0 = good, 3.0 = satisfactory, 4.0 = sufficient). Most students (77.7%) reported having no previous university experience.

There were no significant differences in gender (69% female; *χ*^2^(1) = 0.12, *p* = .73), age (*M*_Diff_ = 0.06, *t*(294) =  − 0.15, *p* = .88), self-efficacy (*M*_Diff_ = 0.007, *t*(294) = 0.15, *p* = .88), resilience (*M*_Diff_ = 0.09, *t*(294) = 1.45, *p* = .15), or cognitive self-regulation (*M*_Diff_ =  − 0.002, *t*(294) =  − 0.04, *p* = .97) between students who dropped out of the study and the investigated sample. Those who dropped out differed significantly only in terms of PHQ-9 scores (*M*_Diff_ =  − 1.29, *t*(187) =  − 2.38, *p* = .02). Their PHQ-9 scores were slightly higher (*M* = 6.01, *SD* = 4.95) — which could be a possible reason for dropping out.

### Preliminary Analyses

Center-specific differences in the constructs were evaluated. There were no significant differences between the two centers in terms of depression (T1: *M*_Diff_ = 0.55, *t*(182) = 1.01, *p* = .31; T2: *M*_Diff_ = 0.66, *t*(182) = 0.92, *p* = .36), self-efficacy (T1: *M*_Diff_ =  − 0.06, *t*(182) =  − 1.10, *p* = .27; T2: *M*_Diff_ =  − 0.06, *t*(182) =  − 0.91, *p* = .36), resilience (T1: *M*_Diff_ =  − 0.13, *t*(182) =  − 1.74, *p* = .08; T2: *M*_Diff_ =  − 0.08, *t*(182) =  − 0.92, *p* = .36), or cognitive self-regulation (T1: *M*_Diff_ < 0.001, *t*(182) = 0.02, *p* = .99; T2: *M*_Diff_ =  − 0.03, *t*(182) =  − 0.45, *p* = .65).

### Descriptive Statistics

The mean depression scores were 4.72 (*SD* = 3.72) at T1 and 7.64 (*SD* = 4.84) at T2. The mean scores of the covariates at T1 and T2 were as follows: self-efficacy, *M* = 1.97 (*SD* = 0.39) and* M* = 1.92 (*SD* = 0.41), respectively; resilience, *M* = 2.81 (*SD* = 0.49) and* M* = 2.65 (*SD* = 0.57), respectively; cognitive self-regulation, *M* = 2.84 (*SD* = 0.46) and *M* = 2.65 (*SD* = 0.48), respectively. Moderate correlations between depression and the covariates could be found at both measurement points (self-efficacy: *r*_T1_ =  − .42, *r*_T2_ =  − .45; resilience: *r*_T1_ =  − .49, *r*_T2_ =  − .46; cognitive self-regulation: *r*_T1_ =  − .31, *r*_T2_ =  − .40; all *p* < .01). The intercorrelations were highest between resilience and self-efficacy (*r*_T1_ = .70, *p* < .01; *r*_T2_ = .74, *p* < .01), followed by resilience and cognitive self-regulation (*r*_T1_ = .43, *p* < .01; *r*_T2_ = .52, *p* < .01) and between self-efficacy and cognitive self-regulation (*r*_T1_ = .39, *p* < .01; *r*_T2_ = .52, *p* < .01).

### Changes in Depression

The PHQ-9 scores increased significantly (*t*(183) =  − 9.32, *p* < .001, *d* = 0.69) from *M*_T1_ = 4.72 (*SE* = 0.27) to *M*_T2_ = 7.64 (*SE* = 0.36). When entering medical school, 9.8% (*n* = 18) of the total sample reported ≥ 10 points on the PHQ-9, indicating clinically relevant depressive symptoms. After 8 weeks, the number of students with a PHQ-9 score ≥ 10 nearly tripled (28.8%, *n* = 53).

### Identifying Profiles with Differing Changes in Mental Health

The fit indices for the two-to-six-profile solution are described in Table 1. The LMR test and the entropy criterion indicated that a 2-profile solution fits the present data better than a 5-profile solution. However, the SABIC, BLRT, AIC, and BIC estimates indicated a 5-profile solution. Because of these results, and because better differentiation provided better data interpretability, the 5-profile solution was selected.

**Table 1:** Statistics of profile structures

No. of profiles	LL	FP	SABIC	BLRT (*p*)	LMR LRT (*p*)	AIC	BIC	Entropy
2	− 1003.578	7	2021.489	**0.0000**	**0.0000**	2021.156	2043.660	0.880
3	− 994.194	10	2008.865	**0.0000**	0.1902	2008.388	2040.537	**0.896**
4	− 987.909	13	2002.439	**0.0200**	0.0884	2001.819	2043.613	0.870
5	− 977.826	16	**1988.416**	**0.0000**	**0.0464**	**1987.653**	**2039.092**	0.852
6	− 975.223	19	1989.353	1.000	0.2547	1988.447	2049.531	0.859

*LL* log-likelihood. *FP* free parameters. *SABIC* sample size–adjusted Bayesian information criterion. *BLRT* bootstrapped likelihood ratio test. *LMR* Lo-Mendell-Rubin likelihood ratio test. *AIC* Akaike information criterion. *BIC* Bayesian information criterion. Lower SABIC, AIC, and BIC values and higher entropy levels indicate a better profile solution. The LRT and BLRT indices indicate whether the N-profile solution fits the observed data significantly better than the N-1-profile solution. Significant values are highlighted in bold

**Fig. 1:**
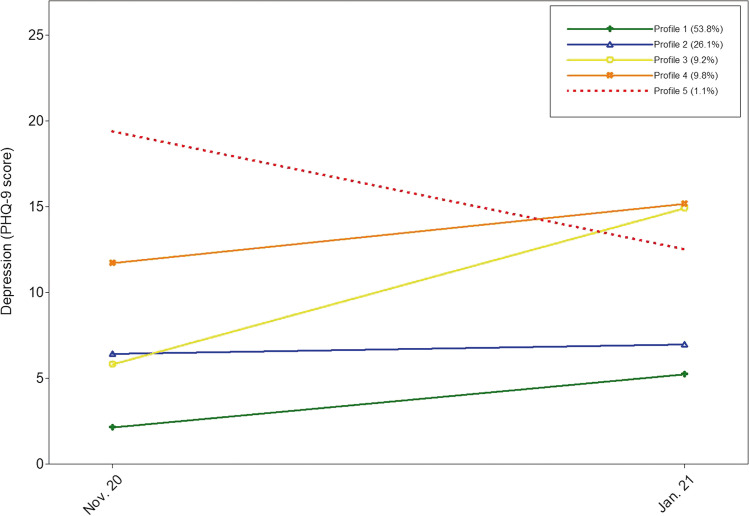
Latent profiles of changes in mental health (measured by depression) and the prevalence (%) of each profile based on estimated posterior probabilities. The lines show the profiles’ mean PHQ-9 scores at T1 (November 2020) and T2 (January 2021). The PHQ-9 scores can be interpreted as follows: 0–4, no depression; 5–9, mild depression; 10–14, moderate depression; 15–19, moderately severe depression; 20–27, severe depression. Profile 1 = no depression; profile 2 = mild depression; profile 3 = depression increase I; profile 4 = depression increase II; profile 5 = persistent depression

Figure 1 displays the five identified profiles. The first profile represented the largest group (53.8%, *n* = 99), showing low PHQ-9 values at both measurement points. Profile 1 was characterized as having *no depression* profile. The second profile was labeled *mild depression*, which represented the second-largest group (26.1%, *n* = 48). Profile 2 was characterized by persistently mild levels of depression. Profile 3 (9.2%, *n* = 17) initially showed mild depression values, which increased to severe levels. We named this profile *depression increase I*. Profile 4 (9.8%, *n* = 18) initially showed severe depression values, which increased even further during the semester. We called this profile *depression increase II*. Profile 5 was the smallest (1.1%, *n* = 2) and showed initially high levels of depression, which decreased during the semester but remained within a clinically relevant range. We labeled this profile *persistent depression*.

### Predictors of Latent Profiles

To investigate the differences among the five profiles regarding relations with self-efficacy, resilience, cognitive self-regulation, and their changes, we included these covariates in the final model by conducting logistic regressions using the automated three-step procedure (R3STEP-command) [31]. The results of the three-step procedure are displayed in Table 2; the *no depression* profile was considered the reference profile.

**Table 2:** Results of the automated three-step procedure (R3STEP): predicting profile membership compared with the no depression profile (profile 1)

	Profile 1 vs. 2	Profile 1 vs. 3	Profile 1 vs. 4	Profile 1 vs. 5
Self-efficacy (T1)	− 0.924**	− 0.960	− 1.913**	− 2.785*
Changes in self-efficacy	− 0.671	− 1.304*	− 1.762***	− 0.750
Resilience (T1)	− 0.847**	− 0.175	− 1.978***	− 3.524*
Changes in resilience	− 0.201	− 0.813	− 0.902*	0.933
Cognitive self-regulation (T1)	− 0.562*	− 1.050*	− 1.416***	− 1.331
Changes in cognitive self-regulation	− 0.275	− 0.985*	− 0.881**	− 0.281

All values are estimates from the R3STEP analysis

*p < .05. **p < .01. ***p < .001

Profile 1 = no depression. Profile 2 = mild depression. Profile 3 = depression increase I. Profile 4 = depression increase II. Profile 5 = persistent depression

Medical students with higher values of self-efficacy and resilience when they entered medical school were more likely to be in the *no depression* group than in the *mild depression*, *depression increase II*, and *persistent depression* groups. Higher initial cognitive self-regulation levels were also associated with a higher likelihood of being in the *no depression* group than in the *mild depression*, *depression increase I*, and *depression increase II* groups. In addition, a decrease in self-efficacy and cognitive self-regulation was associated with a higher probability of belonging to groups with an increase *in depression* (profiles 3 and 4) compared with the *no depression* group. A decrease in resilience was found to be a predictor only for the *depression increase II* profile. The results for profile 5 will not be interpreted because of the small sample size (*n* = 2).

## Discussion

The present study investigated latent profiles of mental health changes (i.e., measured by depression) and their predictive factors among first-semester medical students at two German medical schools. During the periods in which the questionnaire was administered (T1: November 2020 and T2: January 2021), the students attended classes mainly online because of the COVID-19 pandemic. The main findings and their implications are discussed below.

First, we examined overall changes in mental health (PHQ-9 scores) and prevalence rates (PHQ-9 scores of ≥ 10) during the first semester of medical school. The results revealed that after only 8 weeks in medical school, PHQ-9 scores increased with a moderate effect size from PHQ-9 values indicating “no depression” to PHQ-9 values indicating “mild depression.” Examining a student cohort studying under “normal” conditions, Schindler et al. [32] found a comparable significant increase in depression during the first semester. Moreover, our 29% depression prevalence rate at T2 corresponded to the prevalence rate 28% among first-year medical students observed in a single-center study by Ludwig et al. [33], which was also conducted under “normal” study conditions. Given that two previous representative German studies found that medical students were less affected by the COVID-19 pandemic than other students [7, 8], rather than the virtual study conditions, explanatory factors for medical students’ increase in depression levels might be the challenging process of adjusting to the study program, its academic demands, and the highly competitive study environment [32]. The alarmingly high rate (29%) of students with clinically relevant PHQ-9 scores at T2 in this study suggests an urgent need for preventive measures as early as the first semester.

Guided by the assumption that not all students develop depressive symptoms, to better understand vulnerable subgroups, we identified five different depression development profiles (according to the PHQ-9 scores at T1 and T2): First, about half of the students (*no depression profile*, 53.8%) entered medical school with low PHQ-9 values, which remained low during the first semester. These students did not experience significant changes in mental health and thus seemed not to need support, at least in their first semester. Since this profile represented the mentally healthy subgroup, it was used as a reference profile for further analyses. Second, 26% showed persistent mild depression scores that were not yet clinically relevant, as measured by the PHQ-9 (*mild depression profile*). In this profile, no changes in mental health seemed to have occurred because of medical school. Nevertheless, these students should be considered in designing preventive measures to counteract the manifestation or worsening of their mental health status later in medical school. Third, a smaller subgroup (*depression increase I*, 9.2%) entered medical school with low PHQ-9 scores but showed a significant increase in depression in the first semester (*depression increase I*). These students might benefit from primary prevention interventions implemented early in their studies. Fourth, in another profile, deterioration in mental health (*depression increase II*, 9.8%) was identified. In contrast to the *depression increase I* profile, these students had entered medical school with significantly increased PHQ-9 scores. Thus, these students required secondary or tertiary preventive measures. Fifth, this also applies to the *persistent depression* profile (1.1%). Only two students were assigned to this profile already initially indicating severe depression symptoms, which improved slightly during the semester but remained within a critical range, as measured by the PHQ-9. The reasons for slight improvements in these students could not be determined based on the available data. Therefore, because there were only two students in this group, the results regarding the persistent depression profile were treated with caution.

The results for the vulnerable profiles, *depression increase II* and *persistent depression*, are in line with another study [34], showing that some students had entered medical school already having unfavorable mental health status. It is crucial to identify and support such students as early as possible because they have a tendency to be reluctant to seek help [3]. Because the personal identification of students with elevated depression levels is restricted by data protection, low-threshold offers need to be provided (e.g., psychiatric-psychotherapeutic consultation). Silva et al. [10] found a “depression recovery” cluster in the course of medical studies. It is possible that for some students in the *depression increase I* and *depression increase II* profiles, the deterioration in their mental health reflected difficulties in the adjustment process, which regulate themselves later during the study program.

After identifying the profiles, we performed predictor analyses, which revealed that students with higher values of self-efficacy, resilience, and cognitive self-regulation when they entered medical school were likelier to be in the *no depression* group than in the *mild depression* and *depression increase II* groups. However, these findings did not apply to the *depression increase I* profile. Here, only lower values of cognitive self-regulation were a predictor for latent profile membership. Initial values of self-efficacy and resilience did not predict *depression increase I*. The reason for this finding could be that the two profiles showed little difference in terms of mental health at the beginning of medical school. In addition, decreases in self-efficacy and cognitive self-efficacy were associated with a higher probability of being in groups with an *increase in depression* (*I* and *II*) compared with the *no depression group*. A decrease in resilience was found to be a predictor only for the *depression increase II* profile.

Our results regarding self-efficacy are consistent with previous research demonstrating a strong association between self-efficacy and mental distress among college students [16]. The findings of our study showed that this association also applied to medical students and therefore seemed relevant in preventing depression in medical school. High self-efficacy allows individuals to believe that they can control potential threats in their environment and thus affects the perception of stressors and behavior [35]. A review of studies on university students’ self-efficacy found that self-efficacy levels depended on the learning setting and could increase through interventions or curricular changes [36]. Accordingly, the authors made numerous recommendations for promoting self-efficacy in higher education teaching, such as implementing peer teaching offers in the sense of model learning and sensitizing lecturers to provide positive feedback (i.e., verbal persuasion). These measures may also provide initial guidance for medical students, although it is still unclear, whether there are population-specific effects.

Our findings also support the assumption that resilience is a successful process in adapting to adversity and stressful circumstances [37]. Students in the *no depression* profile appeared to adapt well to their studies, whereas students in the *mild depression*, *depression increase II*, and *persistent depression* profiles may have experienced adaptation challenges because they started medical school with lower levels of resilience. Contrary to our expectations, resilience and changes in resilience did not affect profile 3 membership (*depression increase I*). It is possible that the students in profile 3 did not differ in their initial resilience compared with those in profile 1, and that, in contrast to the large increase in depression, changes in resilience occur with a delay. Our findings suggest that students belonging to the *mild depression* and *depression increase II* groups might benefit from interventions to promote resilience. Existing interventions in healthcare professionals are heterogeneous in terms of duration, scope, and format (e.g., resilience workshops, mindfulness training, and mentoring programs) and vary in their effectiveness [38]. Despite increasing efforts to implement resilience promotion programs in medical education, there is still a lack of standardized and evidence-based interventions that can be implemented by medical faculties [39]. A meta-analysis of resilience interventions among higher education students confirmed their effectiveness in reducing stress and depressive symptoms but pointed to the need for larger-scale studies with more robust methodologies [19].

Cognitive self-regulation is considered a relevant skill in dealing with a high workload [21]. However, there is little evidence of its influence on medical students’ mental health. Our findings suggest that cognitive self-regulation abilities are relevant in the context of depression vulnerability. Higher initial cognitive self-regulation levels were associated with a higher probability of belonging to the *no depression* group than to the *mild depression*, *depression increase I*, and *depression increase II* groups, and a decrease in cognitive self-regulation was associated with a higher probability of belonging to groups with an *increase in depression* (i.e., profiles 3 and 4). A potential reason for the decline in cognitive self-regulation could be that cognitive regulation strategies that were effective in previous school learning were no longer effective in the university context and therefore required readjustment. This assumption is also supported by the fact that a large part of the investigated sample (78%) had no prior university experience. Cognitive self-regulation in terms of self-regulated learning can be promoted by devising learning plans and setting goals [40].

Our study has several strengths. It is the first study using an LPA-approach to examine depression development profiles and associated factors in the first semester of medical school. This methodological approach may also be useful in studying other populations. Our results highlight the fact that students differ in terms of vulnerability to developing depressive symptoms. These different vulnerability groups must be considered when developing preventive measures — taking into account the challenge of how to reach those student — as they have different needs. We state that study conditions must be created through curricular adjustments that promote the identified protective factors. Since stressors that occur in the first semester affect all students, our findings may also be relevant to non-medical students.

Nevertheless, several factors limit the generalizability of our results: Our study was based on a moderate sample of students from two German medical schools. The total dropout rate was 38% and students who dropped out of the study had slightly higher depression scores, which may have distorted the profile prevalence rates. It is possible that the actual number of vulnerable students was higher than demonstrated in our results. Moreover, the PHQ-9 is a self-reported instrument that measures the occurrence of depressive symptoms that last at least for 2 weeks. Despite its high specificity and sensitivity to major depressive disorder [23], this instrument does not replace a psychiatric diagnosis. Furthermore, depression is a construct with multifactorial underlying influences [13]; therefore, influences external to medical school cannot be excluded. Moreover, since the data were collected during a predominantly virtual semester, the results can be interpreted only in this context. Although other studies have demonstrated similar increases in depression and prevalence rates in offline contexts [32, 33], influences of COVID-19 and the online environment cannot be excluded entirely. Furthermore, we examined only two measurement points in the first semester. Therefore, no conclusions can be drawn regarding whether the observed changes in mental health were short-term fluctuations or long-term changes that may manifest themselves, and further research is needed to address this issue. Our results should be replicated with a larger sample under “normal” study conditions.

Based on the results of our study, we conclude the following: First, person-centered analyses of medical students’ mental health help to better understand medical students’ varying depression vulnerabilities, their prevalence, and their predictive factors. Second, preventive measures should be implemented in the first semester of medical curricula to address the needs of different subgroups of vulnerability. The promotion of self-efficacy and cognitive self-regulation might be beneficial to all vulnerable groups. Vulnerable groups with *mild depression*, *depression increase II*, and *persistent depression* additionally might benefit from measures aimed at strengthening their resilience. In addition, low-threshold support services should be established for students who enter medical school with elevated levels of depression. Third, further research is needed to explore how to reach the vulnerable subgroups and how the identified protective factors can be promoted through curricular adaptations (behavior-oriented and structural prevention).

## Data Availability

The datasets generated and/or analyzed in the current study are not publicly available due to privacy and data storage restrictions on university internal servers. The datasets are available from the corresponding author upon reasonable request.
